# 2141: What is the role of race and ethnicity in the development of thionamide-induced neutropenia?

**DOI:** 10.1017/cts.2017.246

**Published:** 2018-05-10

**Authors:** Iric R. Guthrie, Mark D. Ehrhart, Jose R. Bucheli, Mark R. Burge

## Abstract

OBJECTIVES/SPECIFIC AIMS: Thionamides are anti-thyroid drugs (ATD) that are commonly used to treat autonomous thyrotoxicosis. Although efficacious, these medications carry a risk of neutropenia or agranulocytosis in a small but finite proportion of the patients who receive them. Some risk factors for thionamide-induced neutropenia have been identified, including body mass index (BMI) and dose, but the role of race and ethnicity in the pathogenesis of this potentially life-threatening side effect is not known. We hypothesize that there will be no effect of race or ethnicity on the change in absolute neutrophil count (ANC) following initiation of thionamide therapy among adult patients with thyrotoxicosis. METHODS/STUDY POPULATION: Data from the electronic medical record at UNM HSC were obtained using a standard database query for the years 2000–2016. Inclusion criteria were the prescription of an ATD, an ANC recorded within 30 days of initiating ATD therapy (pre-ATD), and an ANC recorded between 75 and 365 days after starting an ANC (post-ATD). Patients taking other agents known to cause neutropenia and agranulocytosis, such as clozapine, allopurinol, or chemotherapy, were excluded. Patients were assigned to racial and ethnic groups as follows: Hispanic, non-Hispanic Caucasian (NHC), native American, Black, and Asian. The post-ATD ANC was defined as the nadir ANC observed after the ATD was started. “Delta ANC” was defined as [(post-ATD ANC)−(pre-ATD ANC)]. ANOVA analysis with Bonferroni-adjusted post-hoc testing was performed to examine differences in the mean changes of ANC across ethnic groups. RESULTS/ANTICIPATED RESULTS: In total, 123 adult patients met inclusion and exclusion criteria and were included in the analysis. No significant difference was found between any of the racial groups with regard to age, sex, BMI, pre-ATD ANC, or the pre-ATD to post-ATD ANC interval. The native American group showed a significantly greater post-ATD ANC (not shown) and Delta-ANC as compared with the other groups. Delta ANC Hispanic=−1.4±3.3, Caucasian=−0.6±3.3, Black=−0.9±4.1, Asian=−3.8±4.8, native American=3.6±5.1 (all units per mm^3^; *p*<0.001). DISCUSSION/SIGNIFICANCE OF IMPACT: In this cohort of New Mexicans with thyrotoxicosis, native American race was protective against thionamide-induced neutropenia.

